# Agentic Artificial Intelligence in Medical Imaging Education: Architectural Autonomy and the Risk of Cognitive Surrender

**DOI:** 10.1002/jmrs.70099

**Published:** 2026-05-17

**Authors:** James Hayes

**Affiliations:** ^1^ Virtual Medical Coaching Christchurch New Zealand

**Keywords:** artificial intelligence, clinical reasoning, cognitive surrender, diagnostic complementarity, medical imaging education, Tri‐System Theory

## Abstract

As agentic artificial intelligence systems become increasingly embedded in medical imaging, practice is moving from episodic decision support to workflow‐based architectures that alter how practitioners think and practise. Medical imaging practice is traditionally conceptualised using Dual Process Theory, which describes how practitioners use their System 1 (intuitive decision making) and System 2 (analytic decision making) in practice. However, as more practitioners incorporate agentic artificial intelligence systems into their workflow, a Tri‐System framework may be required. This Perspective paper will show how the practitioner and an agentic artificial intelligence system become part of a cognitive team known as System 3. It will argue that an appropriate level of cognitive surrender should be considered and that current decision making should be reframed through diagnostic complementarity, with added emphasis on structured human and AI interaction to achieve optimal performance. We recommend the implementation of the following educational methods in radiography programmes: (a) training students using fault‐injected medical images to reinforce the importance of human verification in image interpretation; (b) preparing students to supervise the performance of agentic artificial intelligence systems; (c) normalising AI‐assisted activities to mitigate potential deskilling.

## Graphical abstract

Agentic AI systems embedded within imaging workflows may alter reflective reasoning by reshaping when and how verification occurs. This Perspective proposes a Tri‐System framework and argues for educational recalibration to preserve diagnostic complementarity, calibrated trust, and accountable human supervision within architecturally integrated clinical environments.

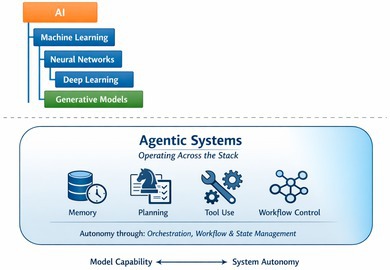

## 1 Introduction

Artificial intelligence (AI) is increasingly being adopted in radiation procedures. Medical Radiation Practitioners are currently utilising AI for image processing, work‐list management, report structuring, and protocol management. Agentic artificial intelligence entails the coordination of action sequences in clinical operations via the integration of elements like memory, planning, tool use, and workflow control, instead of generating individual outputs. The American College of Radiology (ACR), Society for Imaging Informatics in Medicine (SIIM), and Canadian Association of Radiologists (CAR) have also issued position papers on the usage and ethics of AI in radiology [1, 2, 3, 4]. More broadly, the literature describes a trend of increasing “hybridity” between humans and AI in healthcare [5].

In this paper, medical radiation science describes the wider profession that includes both diagnostic radiography, radiation therapy, and nuclear medicine. The term radiography is defined here as the specific practice of diagnostic imaging carried out by Medical Radiation Practitioners. The term Radiographer as used in the UK and Ireland, Radiologic Technologist, as used in the United States of America, and Medical Imaging Technologist as used in New Zealand are considered equivalent to Medical Radiation Practitioner and are included within this scope. Radiology describes the medical discipline practised by Radiologists.

The cognitive environment of professional practice is shifting as AI systems move from individual decision aids to embedded components of organisational workflow architecture [5, 6]. The two systems described in Dual Process Theory (intuition and analytical reasoning) remain central to understanding expertise in medical radiation practice [7, 8]. Research has explored the possibility of an “external cognition” in which AI systems function as a cognitive partner or “third mind” in a triadic cognitive ecology [5, 6].

In such environments, the critical variable is not algorithmic capability alone, but the architectural configuration that shapes when and how reflective reasoning is activated.

Medical Radiation Science educators must distinguish between generative AI tools that produce text or image outputs and agentic radiology AI architectures that simulate autonomous, clinically knowledgeable behaviour. This Perspective recommends that a clear pedagogical distinction is required between the two types of applications in radiography education to support necessary pedagogical revisions.

Educational navigation in the context of AI requires more than learning about AI applications. Graduate competencies must be redefined. The key question is not whether AI will replace medical radiation practitioners in the imaging department but how we reorient our pedagogical thinking concerning reasoning, supervision, and accountability in the context of systems that are architected around AI.

The aim of this perspective is to examine how agentic AI systems alter the cognitive architecture of clinical decision‐making in medical imaging, and to propose a Tri‐System framework to inform curriculum design and professional practice in medical radiation science.

### 1.1 From Generative Models to Emerging Agentic Workflows

Most generative AI systems produce discrete outputs such as summaries, classifications, or parameter suggestions. In imaging, generative AI may assist with structured reporting, image reconstruction, and protocol selection [5, 6]. In each of these cases, the output of the system remains visible and can be independently reviewed by the user before passing it on to the next step or activity in the workflow. This provides a clear opportunity for human review.

Integrated agentic workflows extend beyond discrete outputs to coordinate sequences of actions across clinical systems. Such architectures may integrate patient history, prior examinations, protocol databases, scheduling systems, pre‐configuring protocols, adjusting parameters, completing reporting templates, and updating queues before human review occurs.

In architectural integration, the change is one of process rather than of intelligence. The role of the practitioner shifts from entering discrete inputs to supervising a process synchronised with the clinical workflow.

**FIGURE 1:**
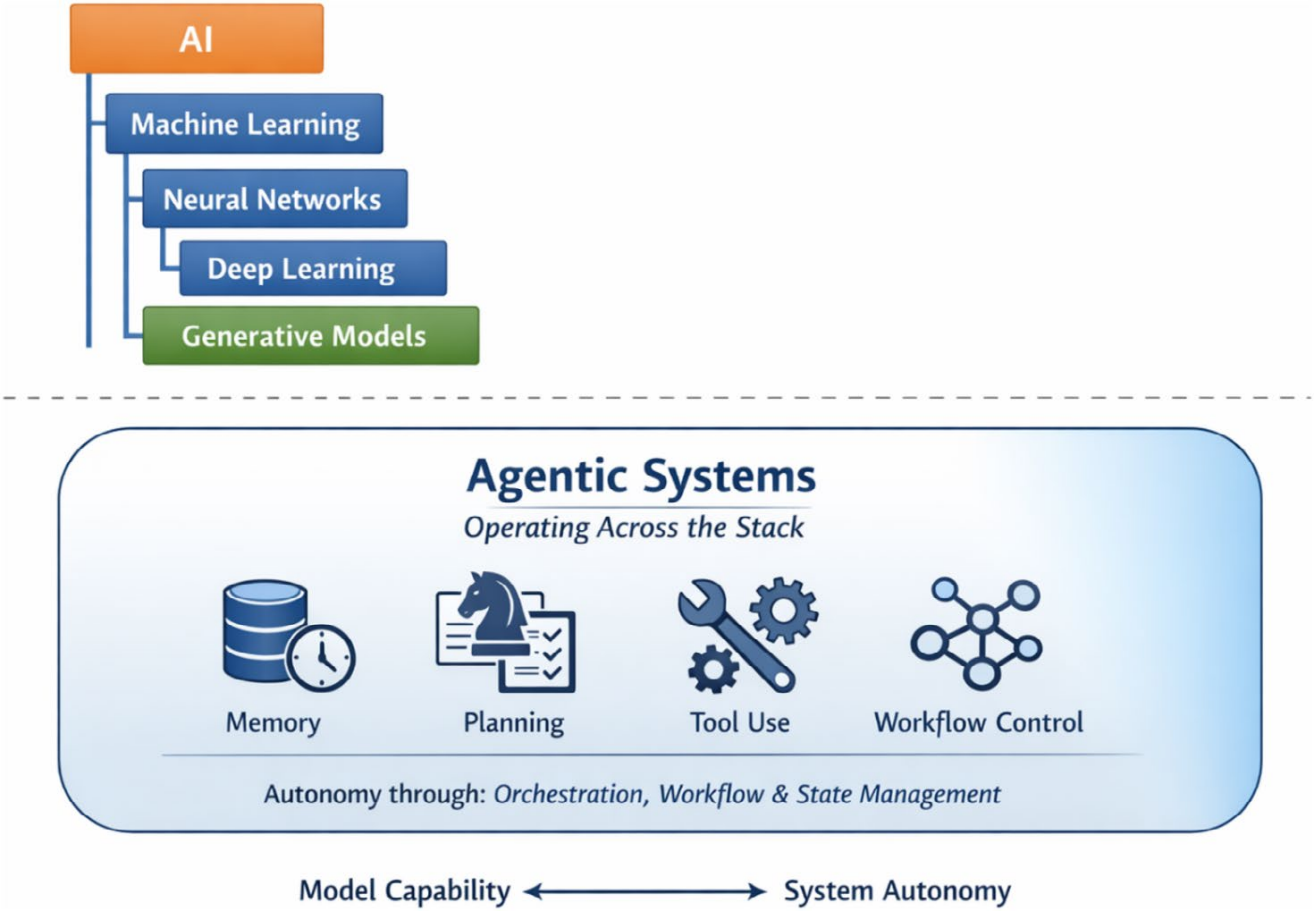
Relationship between AI model capability and agentic system architecture.

Figure 1 illustrates the context for contemporary generative models and agentic system architectures. Deep learning models within the machine learning stack produce outputs such as classifications, image reconstructions, parameter suggestions, and structured reports [5, 6]. Andrew Ng of Stanford University noted that an agentic system is not a form of higher‐level AI that is more intelligent in the sense of being able to reason better or understand what is going on. Rather, it is one layer of abstraction above the capability of the model itself and combines one or more models with other components such as working memory, planning, tool actions, and workflow management to achieve autonomy (personal communication, 14 March 2026). In other words, the model provides the capability, and the system architecture that contains it provides the autonomy. This concept is important for medical imaging education, as it is the transformations happening in the clinical workflow that are changing the way practitioners engage with the decision‐making process.

In the context of a radiographic process, the individual elements of an agentic system have distinct functions. The memory function allows the system to store past imaging, patient information, and protocols used throughout the workflow process. The planning function involves organising the sequence of the processes that include the selection of protocols, adjustments of parameters, and reporting. Tool use refers to interaction with clinical systems such as Picture Archiving and Communication System (PACS), Radiology Information System (RIS), and modality consoles. Workflow control regulates the sequence of workflow tasks, often before human intervention occurs.

These processes differ from traditional AI systems that produce results in a single step and allow a clearer point for review. By coordinating multiple actions across the workflow, agentic systems may reduce natural pause points for reflective thinking, increasing the risk of cognitive surrender.

The implications for education are substantial. We can no longer teach only interpretation of AI outputs; graduates must coordinate AI‐mediated processes, recognise unforeseen dependencies, and intervene appropriately within the decision chain. Curriculum design must shift from focusing primarily on technical application toward developing competence in managing and overseeing integrated system architectures and associated activities.

### 1.2 Cognitive Calibration: Tri‐System Theory and System 3

For decades, the dominant theoretical frameworks have been dual‐process models of reasoning that describe how clinicians process information in the healthcare environment [7, 8]. This Perspective extends that framework by incorporating a Tri‐System model in which an extrinsic “third” system, referred to as System 3, represents artificial cognition composed of external, technological, and data‐driven reasoning capabilities operating within the clinical information flow. Hence, different forms of information may be foregrounded or taken for granted within the decision environment.

Within a Tri‐System framework, medical radiation education must prepare graduates to think clinically while recognising the influence of artificial cognition in shaping perceived features and restricting available options. Curriculum objectives should therefore ensure that graduates exercise metacognitive monitoring of System 3 and engage in reflexive reasoning deliberately rather than incidentally.

### 1.3 Cognitive Surrender

In AI terminology, cognitive surrender is the act of accepting and adopting the output of a cognitive aid without carrying out a careful examination or reflection [9]. This phenomenon differs from automation bias, which describes the tendency to rely on automated systems over one's own judgement [10]. In architecturally integrated environments, outputs may be embedded within workflow in ways that reduce salient breaks for cognitive scrutiny, leading to unexamined acceptance.

Evidence demonstrates that both physical and educational environments influence human cognition and behaviour, including in medical imaging education and simulation‐based training contexts [11, 12, 13, 14]. Research in medical imaging education has examined immersive virtual reality (VR) simulation, with randomised and cohort studies demonstrating improvements across multiple clinical performance variables compared with traditional non‐immersive methods [11, 12, 13, 14]. For example, Karimi et al. demonstrated improved clinical preparedness among radiography students trained using immersive virtual reality compared with traditional simulation methods [14]. Immersive VR training in radiation safety has demonstrated greater reductions in occupational exposure compared with didactic education [15, 16, 17]. The radiography education literature therefore supports the broader proposition that structured simulation, feedback, and continuity between training and performance assessment can influence learner behaviour, clinical performance, and preparedness [12, 13, 14]. This may be relevant to reducing cognitive surrender in AI‐mediated clinical workflows, although this relationship requires direct empirical investigation.

### 1.4 Human Factors Considerations

Humans may follow automated decisions they believe to be more technically capable than their own [10]. At the same time, they can switch to alternative sources of decision‐making upon detecting an error in the current automation [18]. The educational challenge is therefore to calibrate learners' reliance on decision‐making sources appropriate to the clinical context.

In this context, students must train verification behaviour in a structured way. If architectural integration eliminates natural checkpoints, artificial checkpoints must be introduced for training purposes. In simulation, assessments and learning reports must reintroduce the friction that has been removed, training students to engage System 2 deliberately rather than only in obvious situations.

Diagnostic complementarity refers to a condition in which errors in human and AI diagnostics are only partially coincident, allowing each to check and enhance the other's performance [19]. Complementarity will be maintained only if meaningful human involvement is retained in the combined system.

Table 1 summarises the interplay between Systems 1, 2, and 3 within imaging workflows. It illustrates how artificial cognitive processes reshape traditional dual‐process reasoning and identifies where clinical judgement may become diminished or non‐deliberate.

**TABLE 1:** Tri‐System Theory applied to radiography practice.

Concept	Dual‐process context	System 3 impact	Risk to professional judgement
System 1	Fast, intuitive processing	Rapid AI outputs may anchor perception	Superficial confirmation bias
System 2	Slow, analytical reasoning	Reduced pause points may limit engagement	Diminished verification depth
System 3	External artificial cognition	Supplements or supplants reasoning	Cognitive surrender; autonomy erosion
Calibrated Trust	Metacognitive oversight	Requires AI literacy and workflow awareness	Deskilling if unexamined reliance persists

The key consideration is not the model accuracy per se but rather the context of reflective processing. When users must physically interrupt workflow to engage System 2, such interruption may become increasingly difficult. Verification behaviour may remain diminished even when model performance is high, particularly in environments where workflow integration reduces opportunities for deliberate reflective engagement [10, 18].

**FIGURE 2:**
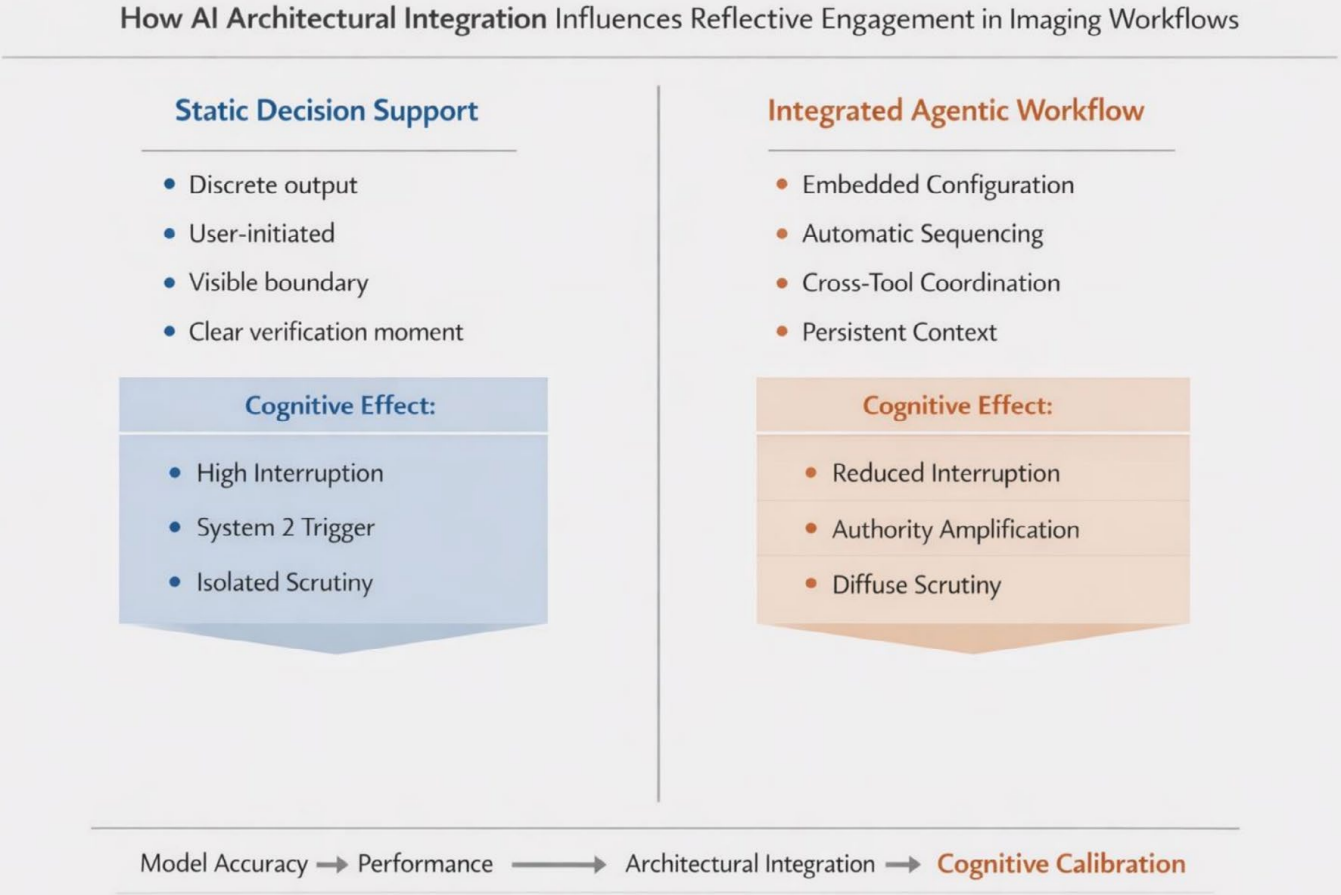
Influence of AI architectural integration on reflective engagement.

To further conceptualise this dynamic, Figure 2 contrasts static decision‐support tools with integrated agentic workflows.

Decision‐making can be supported in two primary ways. The first is to provide a verification point that allows reflection and validation. The second is to embed feedback directly within the decision process. Model quality influences the effectiveness of either approach and shapes user behaviour depending on the availability of reflection triggers.

In static decision‐support systems, users must actively seek advice and define the question they wish to answer. They encounter an identifiable verification point at which the appropriateness of the decision request can be reviewed. In integrated workflows, recommendations are embedded within the decision process, minimising the need for deliberate interruption or explicit question formulation. This has an important impact on the design of reflective oversight mechanisms within architecturally integrated systems.

### 1.5 Why Architectural Integration May Increase Deference

Professional decisions are shaped by the environment in which they are made. In the context of Dual Process Theory, reflective processing modes are usually triggered in response to uncertainty or interruptions. Decision support tools provide locations for discussion points; integrated systems reduce friction and decrease the need for specific points of consultation [10, 18].

The depth of architectural integration can increase the perceived authority of a system, particularly when it integrates information from multiple sources and maintains contextual continuity across system states. The risk is not inherent to the capabilities of the models themselves, but to the cognitive triggers that are introduced through the orchestration process.

### 1.6 Educational Implications of Architectural Integration

The embedding of AI within clinical workflows alters the structure of professional practice. As a result, medical radiation programmes should reconsider core competencies in the following areas:

- Monitoring an automated workflow and ensuring effective human involvement in cases of technology failure.
- Recognising potential sources of latent errors and unintended consequences within technological systems.
- Recording overrides and/or decisions, recognising that technology may not always reach the appropriate conclusion for every clinical situation and ensuring that practitioners are competent to withhold or modify recommended actions when necessary.
- Communicating the rationale of decisions to colleagues and patients, to ensure transparency about why certain actions were performed by the technology‐assisted team.
- Recognising that professional judgement is not eliminated but redistributed within the clinical workflow. To support the effective introduction of AI technologies, education programmes must develop learners' ability to practise and make judgements in new situations, that is, within a relocated judgement space, and ensure that professionals can maintain professional judgement, relationships and ethics in the new contexts provided by technology [20].

As international survey data on AI in medicine is beginning to emerge, medical students generally appear optimistic about the future role of AI in healthcare. In a large multicentre study conducted in Germany, the vast majority of students expected AI to be “very good for medicine” (*n* = 82, 84%), although a minority expressed concern that AI would take over the role of radiologists in particular [21]. In a national multi‐programme survey conducted in Singapore, radiology residents showed a high level of basic knowledge about current applications and AI terminology. However, they reported that there was a “gaping hole” in that AI was not currently included in any part of their curriculum [22]. In Australia, although most medical students believed that “AI would be good for healthcare”, only a minority could confidently explain the “underlying computing concepts of AI” [23]. Consistent with previous findings, in Palestine, students believed that “AI can help to diagnose certain illnesses” but reported that they had no undergraduate training to prepare them for working in environments using “AI assistance for diagnosis” [24]. Finally, in Ireland, although students were optimistic regarding AI, uncertainty persisted regarding the potential impact on their future roles [25].

While these studies suggest generally positive attitudes toward AI among medical students, there is a clear need to address an education gap where students have had some exposure to AI but are not yet competent to practise as members of a human‐in‐the‐loop environment. The challenge for medical radiation education is therefore one of calibration: providing adequate understanding of the roles of human supervision, the underlying systems, and how imaging professionals can function in complementary roles within an architecturally integrated environment. Similar gaps between exposure and preparedness have also been reported among medical radiation technologists in imaging disciplines [26].

### 1.7 Implications for Radiography Practice

The increasing use of AI in healthcare does not diminish the role of the radiologist. While radiologists and reporting radiographers retain responsibility for diagnostic interpretation, medical radiation practitioners are responsible for image acquisition, protocol execution, and immediate clinical decision‐making within imaging workflows. Ethical and responsible practice still resides with the radiographer and radiologist [1]. Trustworthy AI frameworks emphasise transparency, auditability, and complementarity with clinical judgement rather than replacement [27].

The following scenario describes a CT pulmonary angiography examination in which the CT scanner incorporates an integrated system that automatically pre‐sets protocol parameters. The radiographer reviews the parameters and adjusts if necessary. In this case, the radiographer identifies renal impairment and reduces the contrast dose accordingly. The modification is then documented. In this scenario, the use of AI reduces the workload for the radiographer; however, verification and critical appraisal remain essential prior to contrast administration to ensure patient safety.

In projection radiography, agentic systems can pre‐select certain exposure parameters, taking into account body size and previous studies performed. The medical radiation practitioner dealing with the procedure needs to evaluate the appropriateness of selected parameters with regard to the indication. This may help prevent poor image quality or unnecessary radiation dose.

In interventional settings, an integrated system can adjust certain parameters. However, the practitioner still needs to participate in the process of controlling dose, position, and shielding.

Recent work in the radiography literature has also raised the need for discipline‐specific integration of AI into clinical workflows and education, emphasising the importance of role clarity, workflow understanding, and practitioner engagement with AI‐assisted decision‐making [28].

AI may reduce cognitive load for radiologists by automating some tasks such as repeated selection of parameters for examinations, selecting and displaying prior imaging to streamline reporting [29]. With thoughtful implementation of AI, radiologists may have more time for doctor‐patient interactions and high‐level decision making, and the need to override is simply seen as part of their job, a skill rather than something that needs to be fought [10].

### 1.8 Implications for Education and Simulation

Curricula should start to shift from instruction on the use of AI toward the necessity of AI oversight and supervision [30]. Furthermore, simulation provides an educational setting in which verification actions, potential override decisions, and documentation can be intentionally practised and refined without causing any harm to the patient [31].

This aligns with recent radiography‐specific literature, which has identified the need to move beyond general AI awareness toward structured integration within radiography education and clinical workflow understanding [28].

These learning strategies are compatible with existing theoretical perspectives on learning. Experiential learning helps learners actively participate in the use of AI‐driven workflow by means of simulations. Reflective learning helps develop metacognition among learners, thereby helping them gauge how much they rely on the system's outputs. Learning fault injection helps foster experiential as well as reflective learning activities.

An additional feature of the control architecture is a simulation environment in which predefined AI misconfigurations are introduced. Learners are required to detect anomalies, interrogate system outputs, and implement corrective action. This environment enables evaluation of the learner's ability to determine appropriate levels of human supervision, articulate the rationale for those determinations, and produce clear and complete documentation. Performance metrics should extend beyond speed and accuracy of decision‐making to include explanatory depth and calibrated trust in system outputs.

Redesigning the assessment process is vital. Current competency assessment emphasises speed, accuracy and efficiency of task completion. However, with the transition to architecturally integrated systems, additional competencies must be evaluated, including supervisory depth, rationale for overrides, and reflective engagement with system outputs. Objective structured clinical examinations (OSCEs) should therefore incorporate reasoning transparency, documentation of AI involvement, and explicit rationale for override decisions [32, 33].

Faculty development must accompany curriculum reform. Educators require adequate architectural literacy to understand how such systems affect cognitive triggers, modify verification behaviour, and where responsibilities are relocated. In the absence of this understanding of the underlying systems, teaching risks descending into uncritical promotion or reflexive criticism of AI technologies, neither of which adequately prepares students for accountable clinical practice.

Programmes must incorporate systems literacy within their learning outcomes. Graduates should understand:

- training processes and limitations of machine learning models
- sources of bias and dataset shift
- effects of hyperparameter configuration
- impact of algorithmic decision‐making, user interface design and workflow positioning on perceptual salience and clinical decision‐making pathways.

Taken together, these findings position simulation not only as a tool for learning technical skills, but also for learning supervision in the context of human‐automation interaction in clinical settings.

### 1.9 Governance, Accountability and Disclosures

Governance must extend beyond initial implementation and include regular review and audit of system performance. Architecturally integrated AI systems require scrutiny, as algorithm outputs, clinician modifications, and peer review processes must be examined to ensure safe and valid practice. Comprehensive audit trails enhance transparency, traceability, and accountability in clinical care [10].

Clear delineation of responsibility is essential. Vendor, institutional, and healthcare provider accountability literacy within integrated clinical systems should be identified as a graduate attribute within health curricula. Students require education and awareness about the relationships between vendor design responsibility, institution deployment responsibility, and healthcare provider practice oversight responsibility within integrated systems of care. Therefore, graduates should be given the opportunity to learn and practise the use of audit logs, model output records, and override records within a defensible accountability structure.

Regulatory frameworks reinforce this distinction. The U.S. Food and Drug Administration's (FDA) guidance on clinical decision support software differentiates between assistive tools whose logic can be independently reviewed by clinicians and software that independently drives clinical decisions, with regulatory implications when transparency and interpretability are insufficient [34].

Data governance also needs explicit attention. Maintaining patient confidentiality, managing dataset shift, mitigating bias, and controlling access are essential for the deployment of trustworthy AI systems [1, 2]. Students must understand the implications for ethical and safe practice and be aware of how the deployment of the system impacts data configuration, data provenance and information boundaries.

Model performance alone is insufficient to determine the adequacy of professional education. Architecturally integrated systems alter how imaging professionals decide, verify, and act within clinical workflows. Medical radiation education must therefore be reconfigured to promote governance literacy and cognitive calibration. Students should learn not only how to use AI, but how to monitor, question, and document its application within accountable systems of care. Efficient workflows do not remove the obligation to retain, practise, and refine professional judgement and reasoning skills [10, 27].

## Funding

The author has nothing to report.

## Conflicts of Interest

The author is the founder and CEO of Virtual Medical Coaching and an Associate Editor for the Journal of Medical Radiation Sciences. He is currently completing a Postgraduate Diploma in Computer Science, specialising in Artificial Intelligence, at Stanford University. He uses agentic AI systems in professional workflows and has experience developing agentic AI models. No external party influenced the writing of this piece, and no third‐party funding was received for it.

## Data Availability

Data sharing not applicable to this article as no datasets were generated or analysed during the current study.
